# Using serious games for cardiopulmonary resuscitation training: a meta-analysis and systematic review

**DOI:** 10.3389/fpubh.2026.1726862

**Published:** 2026-02-05

**Authors:** Zongrong Chen, Ying Chen, Lu Zhang, Zhenhua He

**Affiliations:** 1 Emergency Department, Hangzhou Fuyang Hospital of Orthopedics of Traditional Chinese Medicine, Hangzhou, China; 2 School of Medicine and Health, Shaoxing Institute of Technology, Shaoxing, China

**Keywords:** cardiopulmonary resuscitation, education, meta-analysis, serious games, training

## Abstract

**Background:**

Serious games have emerged as an innovative educational approach, holding potential to improve the quality and efficacy of cardiopulmonary resuscitation (CPR) training. The objective of this meta-analysis was to systematically evaluate the impact of serious games on CPR training and educational outcomes.

**Methods:**

We systematically searched eight databases to identify randomized controlled trials (RCTs) that compared the effectiveness of serious games with that of traditional training in CPR education. The literature search was conducted up to September 20, 2025. All statistical analyses were performed using RevMan 5.4 software.

**Results:**

A total of 11 RCTs involving 409 participants in the serious game groups and 388 participants in the control groups were included. We found no statistically significant differences between groups in CPR compression depth (SMD = 0.63, 95% CI: −0.09 to 1.35, *p* = 0.08), compression rate (SMD = –0.04, 95% CI: −0.53 to 0.44, *p* = 0.86), theoretical knowledge scores (SMD = –0.22, 95% CI: −0.96 to 0.51, *p* = 0.55), or practical skill scores (SMD = −0.18, 95% CI: −1.02 to 0.66, *p* = 0.67). Egger regression analysis indicated significant publication bias in theoretical scores (*p* = 0.015) and skill scores (*p* = 0.022) in this meta-analysis. The high heterogeneity observed across studies necessitates cautious interpretation of these pooled results.

**Conclusion:**

Current evidence does not show a statistically significant advantage for serious games over traditional CPR training methods. Importantly, the evidence is also insufficient to confirm their true equivalence. A cautious stance regarding their widespread adoption is therefore warranted. Future research should prioritize high-quality, adequately powered studies with rigorous designs, standardized intervention protocols (e.g., game types, training duration), and unified internationally recognized assessment standards.

## Introduction

As a critical technique in the field of emergency care, Cardiopulmonary Resuscitation (CPR) plays a pivotal role in saving lives (1). Sudden cardiac arrest (SCA) ranks among the leading causes of death globally, claiming a large number of lives each year. Once SCA occurs, the brain and other vital organs suffer irreversible damage due to oxygen deprivation within minutes (2). Timely and effective CPR can significantly improve the survival rate of patients—for every one-minute delay in initiating resuscitation, the survival rate may decrease by 10%. Administering high-quality CPR within the “golden four minutes” secures precious time for patient rescue and substantially enhances the chances of survival (3). However, the global penetration rate of CPR training remains far from ideal. According to an evidence-based study covering 29 countries, the global CPR training penetration rate is approximately 40%, with significant disparities between high-income and low-income countries—high-income countries consistently report much higher training rates (4). In China, the penetration rate of emergency care skills is less than 1%. Each year, over 1.2 million people die from sudden cardiac death (SCD), and 87.7% of these fatal cases occur outside hospitals (5). In China, for instance, emergency skill penetration is below 1%, contributing to a high burden of out-of-hospital sudden cardiac death (5). Even where training rates are higher, effectiveness varies widely, often due to suboptimal training methods, content, or skill retention (6–8).

With the continuous advancement of technology and the renewal of educational concepts, Serious Games—an emerging tool for education and training—have gradually gained application and attention across various fields (9). Serious Games are defined as games that do not prioritize entertainment, enjoyment, or fun as their primary goals. Instead, they utilize game mechanics to achieve non-entertainment objectives, such as education, training, simulation, and therapy. Integrating the expressive techniques of traditional games with scientific principles, they possess characteristics including purposefulness, applicability, interactivity, and scientific rigor. Serious Games offer unique advantages in education and training. They create an interactive learning or training environment, enhancing participants’ sense of engagement and experience while stimulating their interest and motivation in learning (10). By integrating game mechanics with learning objectives, they offer interactive, engaging, and potentially personalized training experiences that can enhance motivation and engagement (11). Additionally, Serious Games can provide personalized learning content and challenges based on trainees’ progress and capabilities, catering to the diverse learning needs of different individuals. These advantages highlight the great potential of Serious Games in education and training, while also offering new insights and methods for CPR training.

However, primary studies on serious games for CPR training have reported inconsistent findings, and the existing body of literature is limited and heterogeneous. Therefore, this study aims to comprehensively and thoroughly assess the effectiveness of serious games in CPR training via a systematic review and meta-analysis, examine their strengths and limitations in this context, and offer a scientific foundation for the further optimization of CPR training approaches.

## Methods

This meta-analysis was conducted according to the Preferred Reporting Items for Systematic reviews and Meta-Analyses (PRISMA) statement (12). Ethical approval and participant consent were deemed unnecessary, as this study was a meta-analysis.

Inclusion criteria followed the PICOS framework: Participants (P): trainees in CPR education, irrespective of occupation. Intervention (I): CPR training using any serious game. Comparison (C): traditional CPR training (e.g., classroom teaching, video-based self-learning, instructor-led practice). Outcomes (O): CPR compression depth, compression rate, theoretical knowledge scores, and practical skill scores. Study design (S): Randomized Controlled Trials (RCTs). We excluded non-RCTs, studies not in Chinese or English, and those with unavailable or incomplete data.

We systematically searched eight databases (PubMed, Cochrane Library, ClinicalTrials, Web of Science, OVID, CNKI, Chinese Science and Technology Periodical Database, Wanfang) from inception to September 20, 2025. Search terms included “serious game,” “cardiopulmonary resuscitation,” “CPR,” “basic life support,” and related phrases. The full search strategy for PubMed is provided in Supplementary file 1. Reference lists of included articles were hand-searched.

The process of literature screening and data extraction in this study was conducted as follows: Two researchers trained in evidence-based methods carried out the work independently. First, duplicate studies were removed using literature management software. Then, an initial screening was performed by reading titles and abstracts to exclude studies that did not meet the inclusion criteria. Subsequently, full texts of the studies that passed the initial screening were reviewed for a second round of screening, and the final included studies were confirmed. If the two researchers had discrepancies regarding a study, they would reach a consensus through joint discussion, or a third researcher would assist in making a ruling to resolve the dispute. The extracted content covered four aspects: basic information of the included studies, such as the first author, year of publication, and country where the study was conducted; basic characteristics of the study participants, including population features, sample size, and types of serious games used in training; specific intervention measures for the experimental group and the control group; and outcome measures.

Two researchers conducted quality assessment of the included studies in accordance with the risk of bias evaluation criteria for randomized controlled trials specified in the *Cochrane Handbook* (13). In case of discrepancies in their assessments, a third researcher would mediate to facilitate reaching a final consensus. The quality assessment covered selection bias, allocation concealment, performance bias, detection bias, attrition bias, reporting bias, and other biases. Each type of bias was rated into one of three categories: “low risk of bias,” “unclear risk of bias,” and “high risk of bias.”

Data analysis in this study was performed using RevMan 5.4 software, with specific analytical methods as follows: First, heterogeneity was assessed via the Q-test and I^2^ statistic. If the test results met the criteria of *p* ≥ 0.1 and I^2^ ≤ 50%, it indicated low heterogeneity among the study results, and a fixed-effects model was then used for the meta-analysis; if the above criteria were not satisfied, a random-effects model was applied instead. For intergroup comparisons of continuous variables, the mean difference (MD) was used for analysis when all studies adopted the same measurement tool. If different measurement tools were used, the standardized mean difference (SMD) was employed. Both effect sizes were presented with 95% confidence intervals (95% CI). In addition, sensitivity analysis was conducted to evaluate clinical and methodological heterogeneity, while a funnel plot was used to assess publication bias in the meta-analysis results. A *p*-value < 0.05 was considered statistically significant for all analyses in this study.

## Results

### Study selection and characteristics

**Figure 1:**
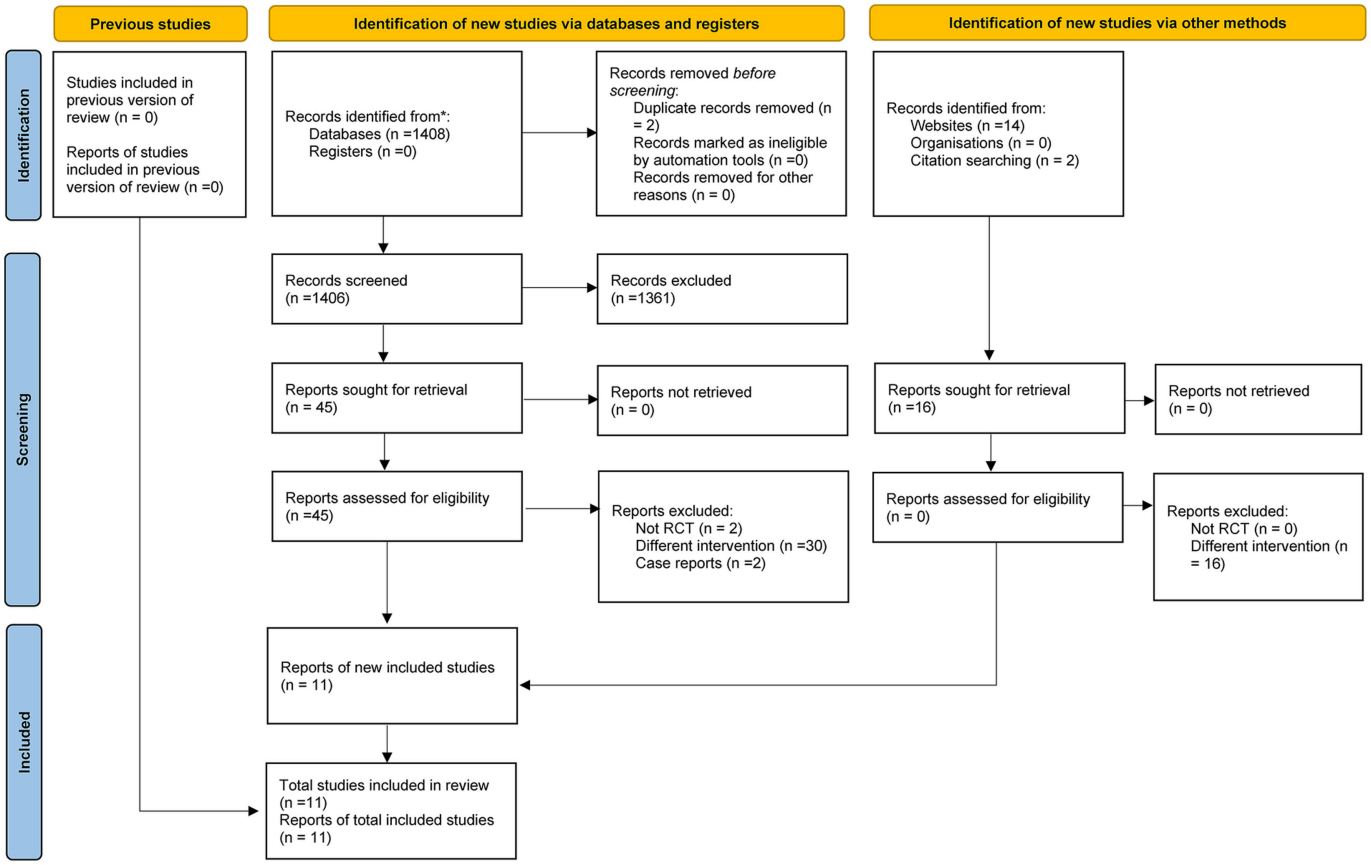
The flow diagram of RCT selection.

The process and results of literature screening for this meta-analysis are as follows: An initial search of various databases yielded 1,408 studies, and 16 additional relevant studies were supplemented through other channels, forming a combined literature pool of 1,424 studies. Subsequently, in accordance with the predefined inclusion and exclusion criteria, an initial screening was conducted by reviewing the titles and abstracts of these studies, leading to the exclusion of 137 studies that did not meet the requirements. The studies that passed the initial screening then underwent a second round of screening via full-text review, during which 34 more studies were excluded. Finally, a total of 11 eligible studies (1, 14–23) were included in this meta-analysis. The detailed literature screening process is shown in Figure 1.

A total of 11RCTs (1, 14–23) investigating the effectiveness of serious games versus traditional methods in CPR education were included in this study. These studies were published between 2013 and 2024, covering 6 countries: Spain, Brazil, France, China, Thailand, and the United Kingdom. The study populations of the included RCTs were diverse, encompassing football coaches, medical students, nursing students, non-medical major college students, senior high school students, and junior high school students. There were variations in sample sizes across the studies, with a total of 409 participants in the serious game groups and 388 participants in the control groups. Regarding intervention measures, the serious game groups adopted serious games as the core teaching method, with forms including dedicated game software (e.g., Staying Alive game, Guess it game), mobile interactive quiz competition games, and gamified training combined with manikins. Some studies also incorporated multimedia courseware or hands-on practice. In contrast, the control groups mainly used traditional teaching methods, such as in-person classroom teaching, online course learning, self-directed learning via multimedia videos, learning through AHA educational CDs, and hands-on practice under the guidance of instructors. Some of these traditional methods focused solely on theoretical learning, while others emphasized skill training. The outcome measures focused on the effectiveness of CPR education, mainly including CPR theoretical test scores, CPR practical skill scores, and specific skill indicators such as compression depth, compression rate, ventilation score, and chest compression interruption time.

### Risk of bias

**Figure 2:**
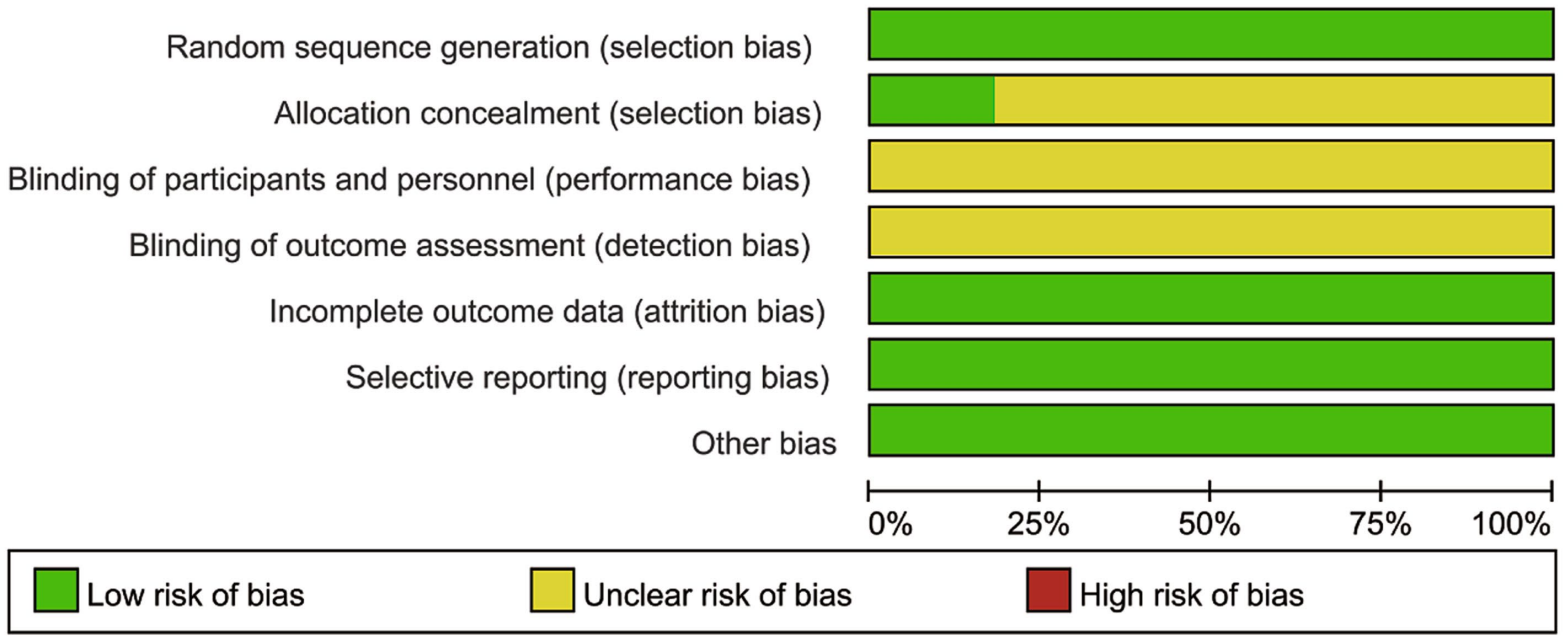
Risk of bias graph.

**Figure 3:**
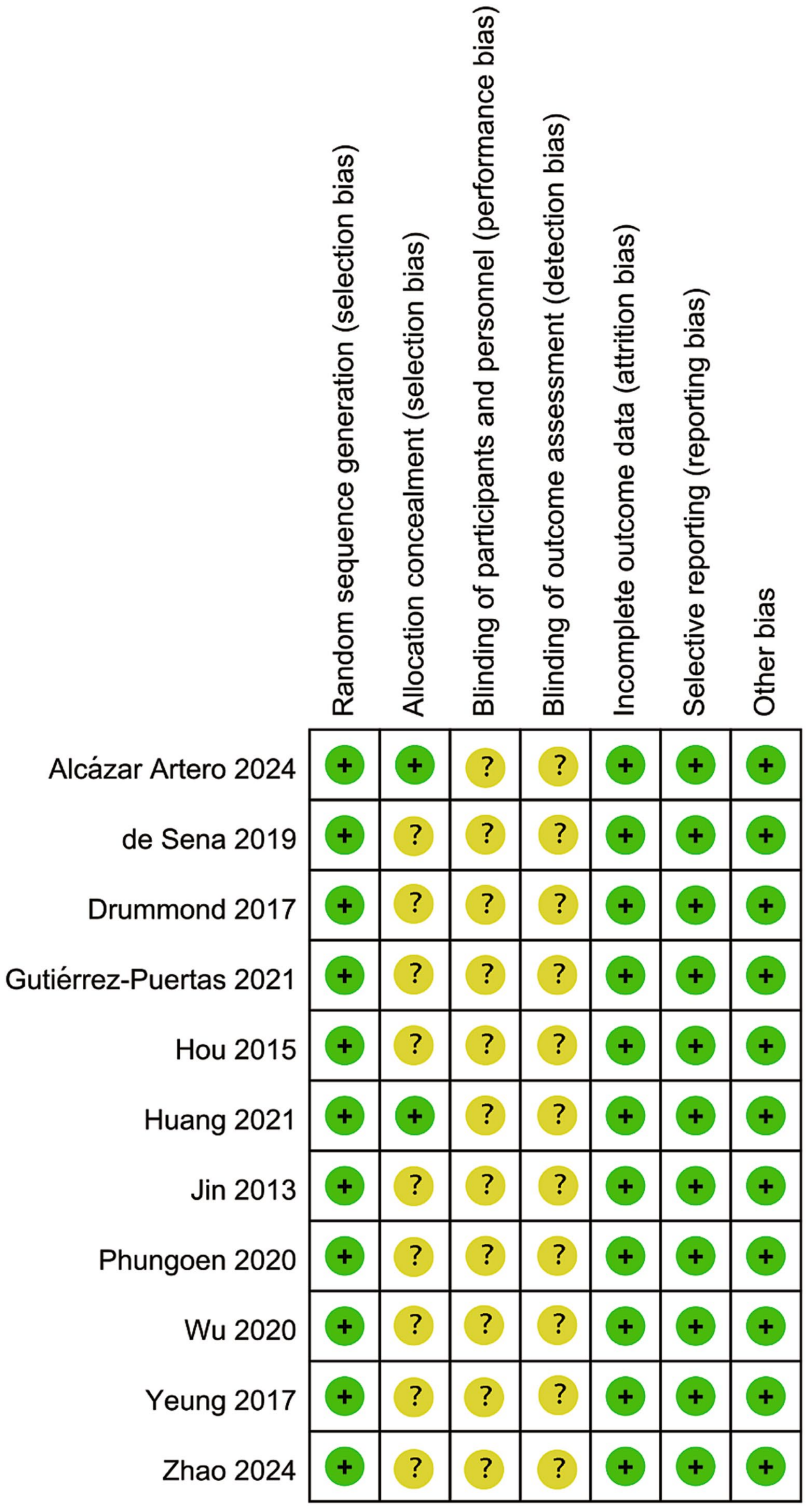
Risk of bias summary (“+” indicates a low risk of bias, “?” indicates an unclear risk of bias, and “-” indicates a high risk of bias).

The risk of bias assessment for the included studies is illustrated in Figures 2, 3, and the overall quality of these included studies was deemed acceptable. Among all 11 RCTs, all reported the generation of random sequences; however, only 2 of these RCTs provided details on the methods used for allocation concealment. No clear information regarding the blinding of participants and researchers was reported across any of the studies. Notably, all 11 RCTs had complete data sets, and no other sources of bias were detected in any of the included studies (Table 1).

**Table 1:** The basic characteristics of included RCTs.

RCT	Country	Study population	Sample size		Intervention		Outcomes
			Serious game group	Control group	Serious game group	Control group	
Alcázar Artero (14)	Spain	Football coaches	31	32	The user wear VR glasses and picks up some haptic controls. The software allows several scenes but in our case the simulation starts in a park, with an unconscious person, with whom a chain of survival must be used	Traditional classroom teaching	The quality, rhythm, and depth of cardiopulmonary resuscitation
de Sena (15)	Brazil	Medical students	23	22	Learning the same CPR content through serious games	Learning CPR knowledge points through online courses	CPR theoretical and skill scores
Drummond (16)	French	Medical students	40	39	learning the CPR content through the Staying Alive game	Learning CPR knowledge points through online courses	CPR compression depth and frequency
Gutiérrez-Puertas (17)	Spain	Nursing student	92	92	Learning CPR knowledge through the Guess it game	Traditional classroom teaching	CPR theoretical scores
Hou (18)	China	Non-medical major college students	35	41	Teaching was delivered through multimedia courseware, CPR manikin practice, and educational game software. Students conducted on-site practical operations for 30 min, with instructors responsible for providing guidance and answering questions.	Teaching was delivered through multimedia courseware, CPR manikin practice. Students conducted on-site practical operations for 30 min, with instructors responsible for providing guidance and answering questions.	CPR theoretical and skill scores
Huang (19)	China	Nursing students	50	50	The serious game group participated in a 4-session cardiopulmonary resuscitation (CPR) educational game experience in a simulated virtual classroom supported by computer-aided simulation. Before the experience, game programmers provided the nursing students with 15–30 min of training and explanation on game operations.	The control group conducted 4 sessions of independent learning in a multimedia classroom, mainly watching CPR-related videos and cases. A theoretical course instructor demonstrated the operation to address the doubts that arose among nursing students during their learning process.	CPR theoretical scores
Jin (20)	China	Medical students	13	18	CPR-related knowledge points and defibrillation operation key points were learned through the physician training software MicroSim installed on computers in the multimedia room	Students self-learned CPR knowledge and skill key points by watching AHA teaching CDs and instructional videos, and practiced skills freely on manikins. During this period, they could also study teaching materials independently.	CPR theoretical scores
Phungoen (21)	Thailand	Medical students	52	53	The traditional teaching mode was used for learning theoretical knowledge, and during the training, students could play the Resus Days game freely on their own mobile phones.	Theoretical knowledge was learned through the traditional teaching mode, and CPR operation practice was conducted under the guidance of instructors.	CPR theoretical scores
Wu (22)	China	Senior high school students	65	54	Cardiac first aid theoretical knowledge was learned through a mobile interactive quiz competition game, and CPR operation practice was conducted using QCPR Classroom manikins.	External chest compression practice was conducted using a compression feedback system.	CPR skill scores, CPR compression depth and frequency
Yeung (1)	UK	Junior high school students	25	27	Students learned CPR first aid only using the Lifesaver (app/software) on tablets, without guidance from professional instructors or practical practice.	A CPR training course with face-to-face instruction by lecturers. Each student takes turns practicing CPR skills on a manikin, with a teacher-to-student ratio of 1:6.	CPR skill scores, CPR compression depth and frequency
Zhao (23)	China	Nursing students	78	50	The equipment for the Serious Game Group comprised Resusci Anne manikins and the CPR Classroom application, which included a built-in racing game. Students were divided into groups, and each group performed external chest compressions on the manikins in a relay race format; the quality of these compressions—encompassing compression depth, compression rate, and proper recoil—controlled the speed of the racing car representing their group, with higher compression quality leading to faster car speed. The racing speed of each group’s car could be monitored in real time on a large screen; the game lasted 2 min, and the ranking and score of each group were displayed according to the order in which their racing cars crossed the finish line.	For the control group, students watched the *Basic Life Support* CD-ROM, while the instructor explained key knowledge points and operational essentials. Following the video and the instructor’s explanations, students practiced while observing—first practicing discrete movements, then the complete CPR sequence—with real-time guidance on their operations provided by the instructor team.	Total score of operational skills, the score of external chest compressions, the score of ventilation, the depth of external chest compressions, the rate of chest compressions, the interruption time of chest compressions, and the time ratio of external chest compressions.

CPR stands for cardiopulmonary resuscitation; AHA stands for American Heart Association.

### Meta-analysis results

CPR Compression Depth: Pooling 4 studies (*n* = 378) under a random-effects model (I^2^ = 91%), we found no significant difference between groups (SMD = 0.63, 95% CI: −0.09 to 1.35, *p* = 0.08), although the point estimate favored serious games.

CPR Compression Rate: Pooling 4 studies (*n* = 378) under a random-effects model (I^2^ = 81%), there was no significant difference (SMD = –0.04, 95% CI: −0.53 to 0.44, *p* = 0.86).

CPR Theoretical Scores: Pooling 6 studies (*n* = 541) under a random-effects model (I^2^ = 93%), no significant difference was found (SMD = –0.22, 95% CI: −0.96 to 0.51, *p* = 0.55).

**Figure 4:**
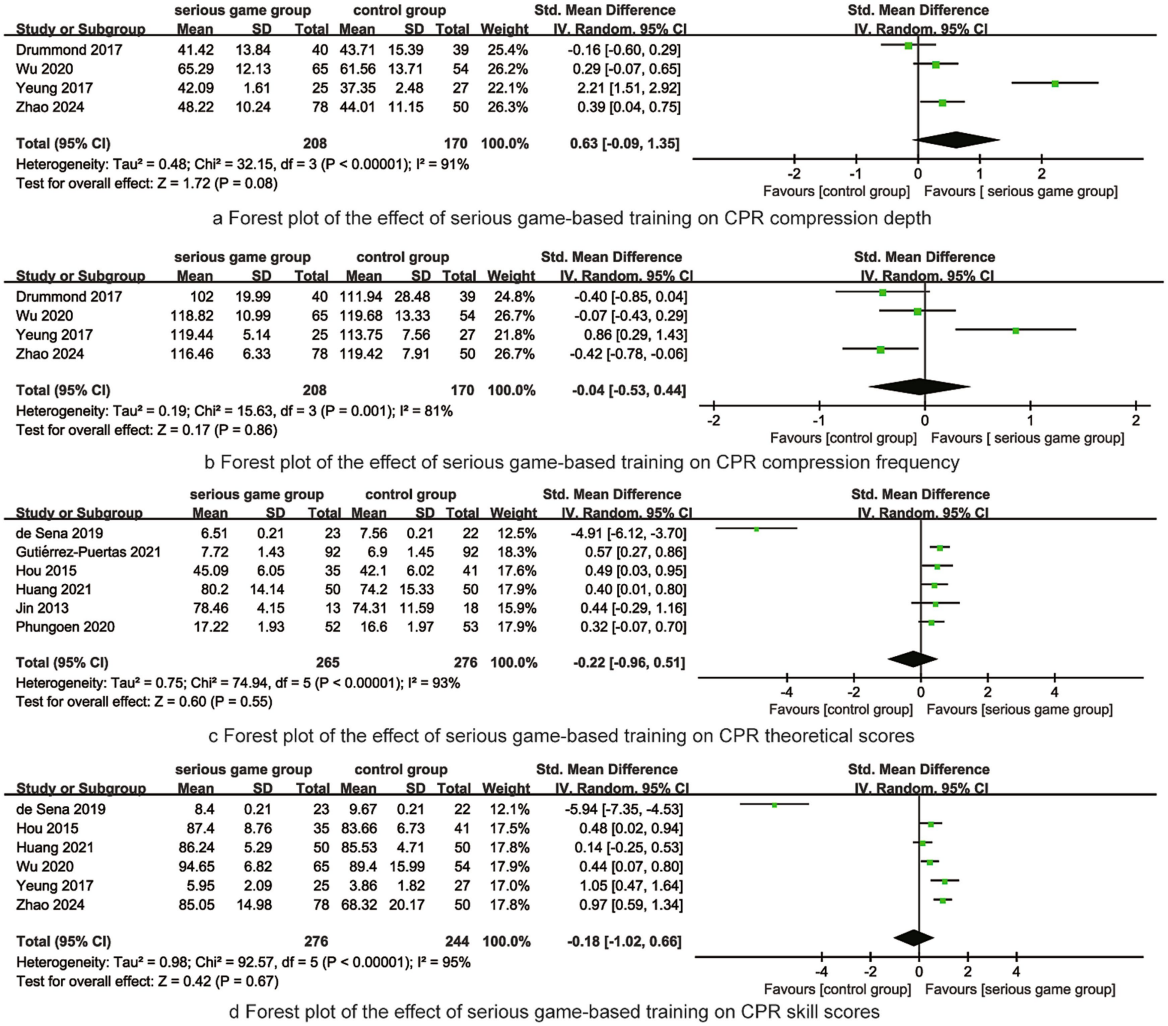
Frost plots of synthesized outcomes.

CPR Skill Scores: Pooling 6 studies (*n* = 520) under a random-effects model (I^2^ = 95%), no significant difference was found (SMD = –0.18, 95% CI: −1.02 to 0.66, *p* = 0.67). Forest plots are shown in Figure 4.

### Publication bias and sensitivity analysis

**Figure 5:**
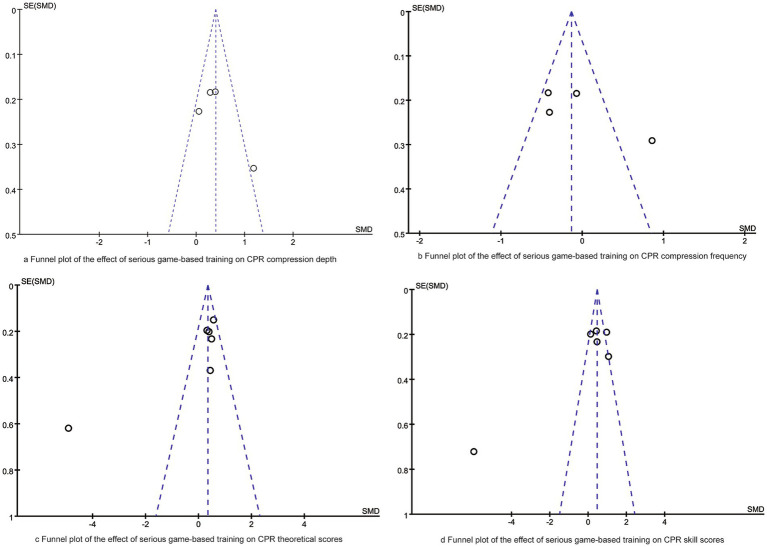
Funnel plots of synthesized outcomes.

Funnel plot analysis was conducted to assess publication bias for each outcome. The funnel plots (Figure 5) for CPR compression depth and compression rate were approximately symmetric, indicating a low risk of publication bias. In contrast, the funnel plots for theoretical scores and skill scores showed obvious asymmetry, suggesting potential publication bias. Sensitivity analysis or further retrieval of gray literature is needed to verify the stability of these results. Additionally, Egger regression analysis indicated significant publication bias in theoretical scores (*p* = 0.015) and skill scores (*p* = 0.022) in this meta-analysis.

We performed separate sensitivity analyses using both fixed-effects and random-effects models to evaluate the SMD and 95% CI for each model. Through a systematic one-by-one study exclusion approach, we noted a reduction in heterogeneity when the study by de Sena et al. was omitted. This finding suggests that the research by de Sena et al. might be a contributing factor to the observed heterogeneity across the included studies.

## Discussion

This meta-analysis found no statistically significant superiority of serious games over traditional methods for improving CPR compression depth, rate, theoretical knowledge, or practical skills. This outcome requires careful interpretation in light of the substantial heterogeneity observed across studies. The variability likely stems from two primary sources. First, methodological differences in study design—including the type of serious game (e.g., immersive VR simulations versus simple mobile quizzes), training duration, and assessment tools—act as significant confounders, diluting the consistency of the pooled effect. Second, the diversity in participant demographics (from football coaches to adolescents) and their consequent differences in baseline knowledge, learning motivation, and responsiveness to gamified instruction introduce additional variance (24, 25). The notably high heterogeneity (I^2^ > 90% for knowledge and skill outcomes) underscores an urgent need for standardization in future research. A critical, unresolved challenge lies in optimally balancing didactic rigor (“seriousness”) with user engagement (“gamification”) in game design (26, 27). Certain interventions may overemphasize entertainment at the cost of learning transfer, while others lack clear pedagogical focus. Furthermore, the use of disparate assessment metrics—ranging from multiple-choice tests to complex skill rubrics—severely limits comparability. Adopting unified, internationally recognized evaluation standards, such as the American Heart Association’s CPR skill guidelines, is essential to enhance the validity and generalizability of future findings.

Serious games demonstrated comparable effectiveness to traditional training methods in improving theoretical and practical CPR scores. The American Heart Association (AHA) has incorporated serious games into its updated resuscitation education guidelines, explicitly stating that virtualized, gamified learning models centered on serious games can exert positive effects on CPR training, including facilitating knowledge acquisition and enhancing CPR skill proficiency (28, 29). In practical CPR training scenarios, theoretical and skill assessments are indispensable components of the teaching process (30). Assessment results not only serve as core bases for evaluating training effectiveness but also act as key indicators of learners’ mastery of CPR knowledge and skills. From this perspective, CPR theoretical scores and practical skill scores directly reflect the teaching quality of CPR training programs (11). Consistent with this, the results of this meta-analysis showed no statistically significant differences in theoretical or skill assessment scores between CPR training using serious games and traditional training programs. This indicates that serious games are non-inferior to traditional methods in helping learners acquire CPR knowledge and improve skill levels, achieving comparable teaching effects.

While serious games did not demonstrate significant improvements in theoretical or practical assessment scores for CPR training, the observed trend toward enhanced compression depth indicates their potential utility in focused skill refinement. Future investigations should prioritize the development of sophisticated “gamified feedback” mechanisms. For instance, integrating real-time performance visualization—such as linking compression quality metrics to in-game progress, as exemplified by Zhao et al. (23)—could heighten learner awareness of movement standards. Such immediate, contextual feedback may prove more effective for motor skill acquisition than the delayed corrections typical of traditional instruction. Furthermore, research must adopt a stratified approach to account for varied learner profiles. Populations such as non-medical university students or secondary school pupils may exhibit greater engagement due to the intrinsic interest of gamified formats. In contrast, medical trainees might derive greater benefit from simulations embedded within authentic clinical scenarios. Conducting subgroup analyses in future studies will be essential to delineate the specific populations for which serious game interventions are most appropriate.

Consequently, it is premature to advocate for serious games as a wholesale replacement for conventional CPR pedagogy. A more measured integration as a supplementary tool is advisable. In practice, gamified elements can be strategically incorporated into hands-on skill drills (e.g., targeting compression depth and rate) to capitalize on their interactive and immediate feedback capabilities, thereby reinforcing procedural memory (31, 32). For delivering foundational theoretical knowledge, however, structured courses—whether in-person or online—remain a more reliable modality. For educational institutions implementing such tools, establishing robust evaluation frameworks is critical to ensure that gamification serves defined pedagogical objectives, rather than becoming an end in itself. Finally, for the research community, the heterogeneity and potential biases underscored by this meta-analysis highlight an urgent need for rigorously designed, multi-center, prospective RCTs with larger sample sizes and pre-registered protocols to generate more definitive evidence.

This study has four limitations that should be considered when interpreting the results. First, this meta-analysis was not prospectively registered in PROSPERO or other relevant platforms. The study design and implementation were initiated prior to our full recognition of the necessity of prospective registration for meta-analyses, which may compromise the transparency of our research protocol and the traceability of our analysis procedures. Second, the literature search was restricted to Chinese and English publications, excluding studies written in other languages. This may have led to the omission of potential relevant evidence, introducing a certain degree of language bias into the synthesized results. Third, the included studies exhibited substantial heterogeneity, primarily arising from multi-dimensional discrepancies in study design: participant populations spanned a broad spectrum from football coaches to junior high school students; serious game interventions varied widely, including VR simulations, mobile competition games, and gamified training combined with manikins; and the measurement tools and scoring standards for outcome indicators were not unified across studies. These differences collectively increased the complexity of result synthesis and interpretation. Fourth, the scope of outcome indicators and the volume of evidence were limited. On one hand, the number of included randomized controlled trials (RCTs) was relatively small; on the other hand, outcome indicators only focused on compression depth, compression rate, theoretical knowledge scores, and practical skill scores, without covering critical dimensions such as long-term retention of CPR knowledge and skills, trainees’ self-efficacy in performing CPR, and other key metrics of compression quality (e.g., completeness of chest recoil, effectiveness of ventilation). Given these limitations, future research should conduct more high-quality, adequately powered RCTs with rigorous study designs. These studies should prioritize prospective registration, standardize intervention protocols (e.g., game types, training duration) and assessment criteria (e.g., adopting internationally recognized CPR skill scoring systems), and expand the scope of outcome indicators to more comprehensively and accurately verify the practical application value of serious games in CPR training.

## Conclusion

This meta-analysis synthesizes evidence from randomized controlled trials on the use of serious games in cardiopulmonary resuscitation training. The results show that serious games did not confer statistically significant advantages over traditional training methods in improving CPR compression depth, rate, theoretical knowledge, or practical skills. Importantly, the current evidence is also insufficient to establish their equivalence to conventional approaches. Therefore, serious games should be approached with caution in CPR training practice and should not be broadly promoted as a substitute for established methods. Instead, their role may be better defined as a supplementary tool within a blended training framework.

The available evidence is constrained by several limitations, including a limited number of high-quality RCTs, generally small sample sizes, and considerable heterogeneity across studies in terms of game design, training duration, and outcome measures. These factors may affect the robustness and generalizability of the pooled findings. Future research should prioritize well-designed, adequately powered RCTs that employ standardized intervention protocols—such as clearly defined game mechanics and consistent training durations—along with internationally recognized assessment criteria, such as the American Heart Association CPR skill evaluation guidelines, to reduce heterogeneity and improve comparability. Expanding outcome measurement to include long-term knowledge retention, learner self-efficacy, and comprehensive metrics of compression quality would further strengthen the evidence. Such rigorously conducted studies are essential to clarify the practical value, optimal use cases, and design improvements needed for serious games in CPR education.

## Data Availability

The original contributions presented in the study are included in the article/Supplementary material, further inquiries can be directed to the corresponding author/s.
