# Supplementary material for: Using serious games for cardiopulmonary resuscitation training: a meta-analysis and systematic review
Source: Front Public Health. 2026 Feb 5;14:1726862. doi: 10.3389/fpubh.2026.1726862 (PMC12916568; doi:10.3389/fpubh.2026.1726862)
Supplement: Supplementary file 1 [file Table_1.DOCX]

**Supplementary File 1: Search Strategy**

1. Data Sources

The following electronic bibliographic databases were systematically searched from their respective inceptions to September 20, 2025:

- PubMed/MEDLINE
- Cochrane Central Register of Controlled Trials (CENTRAL)
- Web of Science Core Collection
- Ovid (Embase, PsycINFO)
- ClinicalTrials.gov
- China National Knowledge Infrastructure (CNKI)
- Wanfang Data
- Chinese Scientific Journal Database (VIP)

2. Search Strategy

The search strategy was designed to identify all relevant Randomized Controlled Trials (RCTs). It combined controlled vocabulary (e.g., MeSH in PubMed, Emtree in Embase) and free-text terms related to the core concepts: (1) "Serious Games" or gamification, and (2) "Cardiopulmonary Resuscitation (CPR)" training. No filters for language or publication date were applied during the initial search. The reference lists of all included studies and relevant review articles were manually screened for additional eligible publications.

3. Full Electronic Search Strategy for PubMed (September 20, 2025)

The search strategy for PubMed is detailed below. This strategy was adapted for syntax and controlled vocabulary for use in the other listed databases.

#1 "Video Games"[Mesh] OR "Gaming, Video"[tiab] OR "Game, Video"[tiab] OR "Video Game"[tiab] OR "Computer Games"[Mesh] OR "Computer Game"[tiab] OR "Games, Computer"[tiab] OR "Gaming, Computer"[tiab] OR "Exergaming"[tiab] OR "Exergames"[tiab]

#2 "Serious game*"[tiab] OR "Educational game*"[tiab] OR "Training game*"[tiab] OR "Simulation game*"[tiab] OR "Digital game*"[tiab] OR "Game-based"[tiab] OR Gamif*[tiab]

#3 #1 OR #2

#4 "Cardiopulmonary Resuscitation"[Mesh] OR "Heart Massage"[Mesh] OR "Resuscitation"[Mesh] OR CPR [tiab] OR "cardiac massage"[tiab] OR "cardiopulmonary resuscitat*"[tiab] OR "chest compression*"[tiab]

#5 "Basic Life Support"[tiab] OR BLS[tiab] OR "Basic Cardiac Life Support"[tiab]

#6 "Emergency Medical Services"[Mesh] OR "First Aid"[Mesh] OR "first aid"[tiab] OR "emergency care"[tiab] OR "resuscitation education"[tiab] OR "emergency skill*"[tiab]

#7 #4 OR #5 OR #6

#8 "Randomized Controlled Trial"[pt] OR "Controlled Clinical Trial"[pt] OR randomized[tiab] OR randomised[tiab] OR placebo[tiab] OR randomly[tiab] OR trial[tiab] OR groups[tiab]

#9 #3 AND #7 AND #8
